# Does enhanced cognitive performance reduce fracture risk? a Mendelian randomization study

**DOI:** 10.18632/aging.205325

**Published:** 2023-12-18

**Authors:** Shaoting Luo, Linfang Deng, Yufan Chen, Weizheng Zhou, Federico Canavese, Lianyong Li

**Affiliations:** 1 Department of Pediatric Orthopedics, Shengjing Hospital of China Medical University, Shenyang 110004, Liaoning, P.R. China; 2 Department of Nursing, Jinzhou Medical University, Jinzhou 121001, Liaoning, P.R. China; 3 Department of Pediatric Orthopedic Surgery, Lille University Centre, Jeanne de Flandre Hospital, Lille 59000, Nord Department, France

**Keywords:** fracture, Mendelian randomization, single nucleotide polymorphisms, cognitive performance, causal relationship

## Abstract

Objective: While observational studies have suggested a link between cognitive performance and fracture risk, the causality and site-specific nature are unclear. We applied Mendelian randomization (MR) to elucidate these associations.

Methods: 147 single-nucleotide polymorphisms (SNPs) tied strongly to cognitive performance (*p*< 5e-8) were selected. We performed MR analysis to investigate the causal relationship between cognitive performance and fractures at specific sites, including the wrist, upper arm, shoulder, ribs, sternum, thoracic spine, lumbar spine, pelvis, femur, leg, and ankle. The primary estimate was determined using the inverse variance-weighted method. Additionally, we examined heterogeneity using the MR Pleiotropy RESidual Sum Outlier test and Cochran Q, and employed MR-Egger regression to identify horizontal pleiotropy.

Results: MR analysis identified a causal association between cognitive performance and fractures at the lumbar-spine-pelvis (odds ratio [OR] = 0.727, 95% CI = 0.552–0.956, *p* = 0.023), and ribs-sternum-thoracic spine sites (OR = 0.774, 95% CI = 0.615–0.974, *p* = 0.029). However, no causal association was found for fractures at other sites.

Conclusions: This study provided evidence of a causal connection between cognitive performance and fracture risk at certain locations. These findings underline the potential of cognitive enhancement strategies as innovative and effective methods for fracture prevention.

## INTRODUCTION

Bone fractures, particularly those in older individuals, are a global public health issue [1]. The Centers for Disease Control and Prevention predict that nearly 30% of people aged ≥65 years will fall annually, with 20–30% of these incidents leading to serious or moderate injuries [2]. These injuries can severely hamper independent living and, in some cases, can be fatal. Fractures have a significant influence on the health, financial stability, and general quality of life in older adults [3].

The established risk factors for fractures include demographic factors such as age and sex, physical characteristics such as bone density, and lifestyle habits such as smoking and alcohol use [4]. In addition to these known factors, recent observational studies have suggested a potential link between cognitive performance and fracture risk [5, 6]. Nevertheless, these preliminary findings require more comprehensive scrutiny to discern the causal nature and site-specific implications of this association.

Cognitive performance, an amalgamated concept that includes memory, attention, and executive functions, plays a critical role in daily activities [7]. Cognitive impairments are linked not only to neurodegenerative diseases such as Alzheimer’s but also to increased risks of falls and consequent injuries [8]. Several studies have proposed a causal pathway from cognitive decline to heightened fracture risk, arguing that cognitive dysfunction may lead to a higher incidence of falls, thereby increasing fracture risk [9]. However, this hypothesis requires rigorous scientific verification to establish whether the relationship between cognitive performance and fracture risk is causal or merely correlative.

To further examine and test this hypothesis, we applied Mendelian Randomization (MR) analysis. MR uses genetic variation as an instrumental variable, enabling inference of unobservable causal relationships in observational studies [10]. MR provides a robust defense against confounding factors and biases in observational studies, offering a more precise depiction of the relationship between cognitive performance and site-specific fracture risk in the present study [11, 12].

With a more nuanced understanding of the causal relationship between cognitive performance and site-specific fracture risk, we can develop better strategies for fracture prevention and management that positively affect health and quality of life [13]. This knowledge is particularly relevant for older adults already experiencing cognitive decline, who might need additional care and assistance to avoid falls and fractures.

In summary, we conducted a two-sample MR analysis using large-scale genome-wide association study (GWAS) data on cognitive performance and site-specific fracture risk. This study aimed to clarify the causal effects of cognitive performance on the probability of site-specific fractures. Unveiling the potential causal relationship between cognitive performance and site-specific fracture risk could revolutionize our understanding of the interplay between cognitive and physical health. Furthermore, this work holds the potential to transform public health policies, pushing towards integrated strategies for health promotion that consider both cognitive and physical wellness. Thus, this study may pioneer a new era of preventative care that reduces the burden of injuries in older adults and enhances the quality of life across this vulnerable population.

## RESULTS

### SNP selection and harmonization results

Following the harmonization of effect alleles across the GWASs of cognitive performance and site-specific fractures, we selected multiple index SNPs for investigation. To genetically predict various fractures, we selected 113, 113, 107, and 117 SNPs for femur and upper arm-shoulder fractures, lumbar spine-pelvis fractures, leg fractures, and wrist and ankle fractures, respectively.

### MR analysis outcomes

We performed an MR study based on genetically projected cognitive performance and specific fracture sites. No heterogeneity was detected, as indicated by the *p*-values of 0.879 and 0.888 derived from the Cochran Q-test values for the inverse-variance weighted (IVW) methods, respectively. Neither MR-PRESSO nor the leave-one-out plot and funnel plots identified any outliers. The results of the horizontal pleiotropy test suggested that pleiotropy was not present, as evidenced by an MR-Egger regression intercept of –0.008, a standard error of 0.014, and a directionality *p*-value of 0.556. Based on these results, the IVW estimates were preferred in the absence of heterogeneity or pleiotropy [14, 15]. Our results demonstrated a potential causal effect of cognitive performance on the risk of lumbar spine-pelvis fracture, a conclusion derived from statistically significant findings (odds ratio [OR] = 0.727, 95% CI = 0.552–0.956, *p* = 0.023).

**Figure 1:**
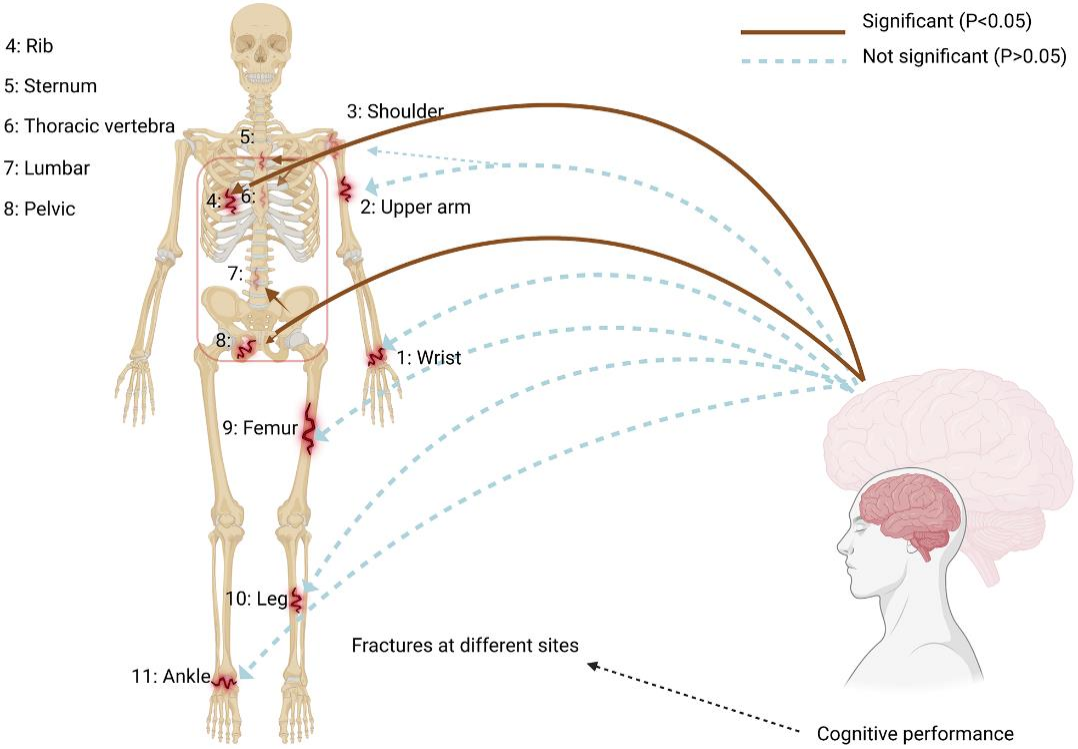
**Impact of cognitive performance on site-specific fractures.** Using a two-sample Mendelian Randomization framework, we showed a causal relationship between cognitive performance and site-specific fracture risk, supporting the existence of a bone-brain axis. The IVW estimate (brown line) is significant (*P* < 0.05). The red box highlights the aspect of cognitive performance that significantly affects fracture incidence.

**Figure 2:**
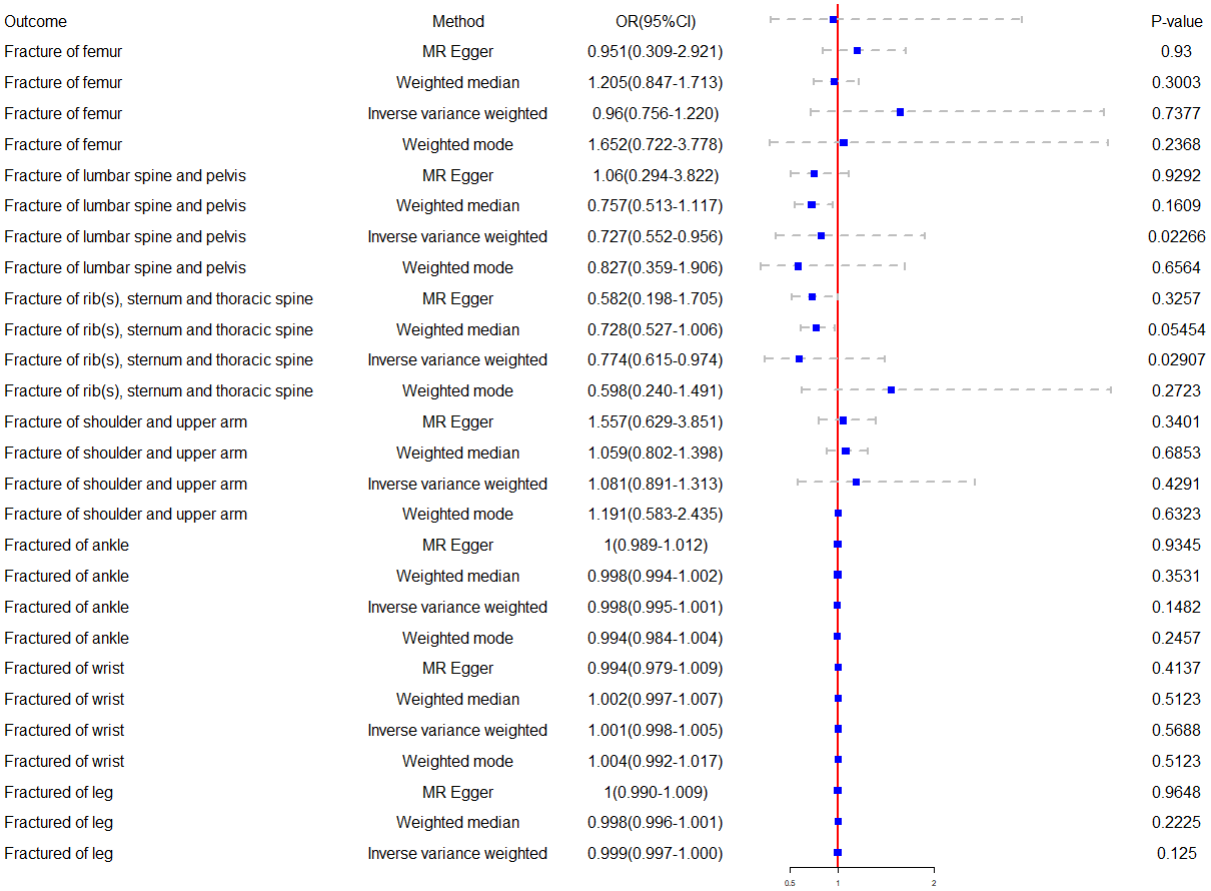
**Forest plot of the Mendelian randomization results.** The causal effects between cognitive performance and site-specific fractures were estimated using different Mendelian Randomization methods.

We further analyzed the causal relationship between cognitive performance and ribs-sternum-thoracic spine fractures, utilizing the MR analysis method. For the latter, we found substantial evidence of a potential causal effect, which showed statistical significance (OR = 0.774, 95% CI = 0.615–0.974, *p* = 0.029). However, our findings indicated no causal relationship between cognitive performance and wrist, upper arm-shoulder, femur, leg, or ankle fractures (Figures 1, 2).

### Horizontal pleiotropy and heterogeneity outcomes

To determine whether the single-nucleotide polymorphism (SNPs) associated with cognitive performance were associated with recognized fracture risk factors, we used the PhenoScanner database. We considered factors, such as aging process, sex identification, fat-free soft-tissue body mass, type 2 diabetes, tobacco use, alcohol intake, and steroid hormone levels. Notably, for aging process and sex identification, the PhenoScanner database did not provide any direct SNP associations. Specific SNPs were linked to each of the following factors: rs9384679 and rs11138947 with body mass; rs73189617, rs62169190, rs11693702, and rs11210871 with smoking; rs11720523, rs2836921, rs10874938, rs11079849, and rs2977464 with alcohol consumption; and rs6860626 with diabetes. However, the consistency of the estimates remained unchanged after removing these SNPs, suggesting that despite accounting for potential risk factors, the causal relationship between cognitive performance and site-specific fractures was not significantly influenced. This was evidenced by our results (β_lumbar_ = –0.304, 95% CI: –0.589 to –0.020, *p* = 0.036) (β_ribs_ = –0.275, 95% CI: –0.512 to –0.038, *p* = 0.023), affirming the robustness of the causal relationship.

**Figure 3:**
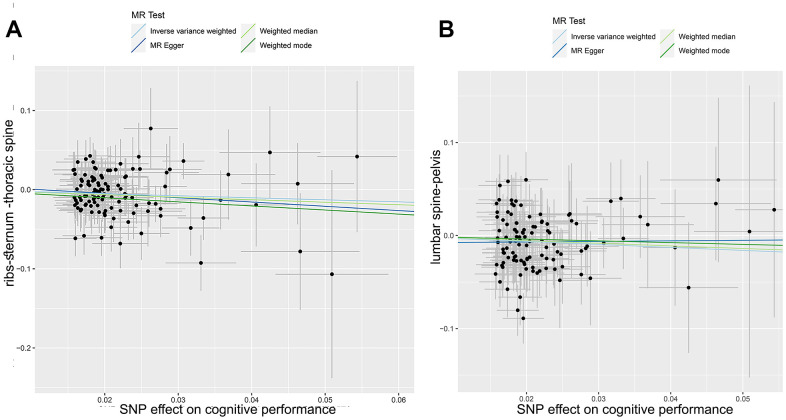
**Mendelian randomization analysis: correlation between cognitive performance and site-specific fractures.** This scatter plot depicts the genetic correlations between cognitive performance and fractures in the ribs-sternum-thoracic spine (**A**) and lumbar spine pelvis (**B**). Different Mendelian Randomization methods were used in the analysis. The slope of each line in the plot indicates the estimated causal effects inferred using each method.

**Figure 4:**
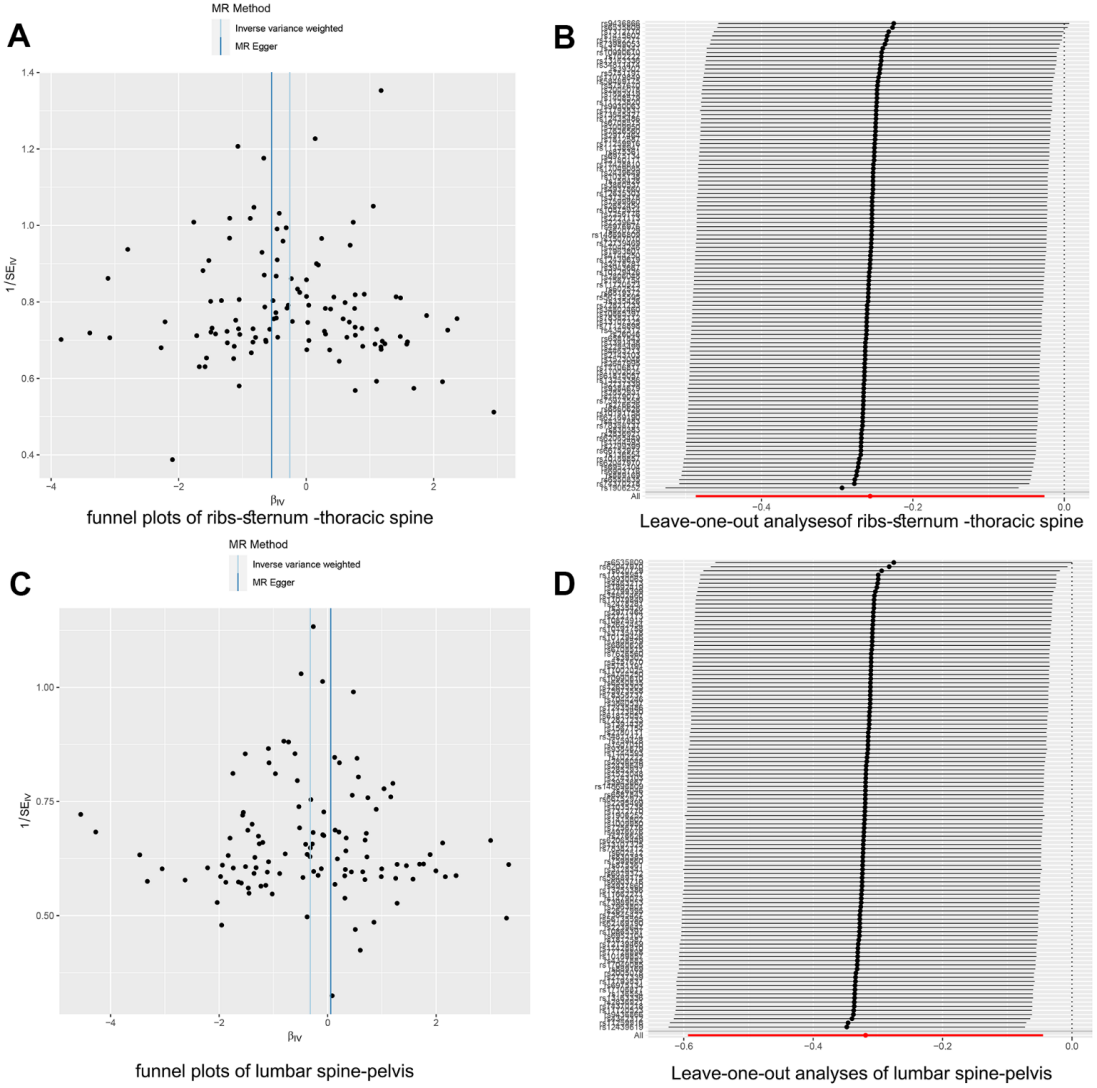
**Two-sample Mendelian randomization heterogeneity test results.** (**A**) The funnel plot for rib-sternum-thoracic spine fractures exhibited a symmetric distribution. (**B**) The 'Leave-one-out' sensitivity test confirmed the robustness of the rib-sternum-thoracic spine fracture results. (**C**) Similarly, the funnel plot for lumbar spine-pelvis fractures showed symmetry. (**D**) The 'Leave-one-out' sensitivity test for lumbar spine-pelvis fractures validated the stability of these findings.

We also employed various methods, such as the MR-Egger regression intercept, leave-one-out analyses, and funnel plots, to test for horizontal pleiotropy for significant estimates. The *p*-values from all MR-Egger intercept tests were >0.05, indicating a lack of horizontal pleiotropy (Figure 3A, 3B). Furthermore, our findings suggested no evidence of pleiotropic heterogeneity, as the derived Cochran’s Q *p*-values were >0.05. This, along with the results of the funnel plots (Figure 4A, 4C) and leave-one-out analyses (Figure 4B, 4D), indicated that the estimates were neither violated nor biased by a single SNP.

### Power analysis outcomes

Finally, we performed a power analysis to evaluate the robustness of our findings. The statistical powers of cognitive performance on the risk of lumbar-spine-pelvis and ribs-sternum-thoracic spine fractures were 0.60 and 0.81, respectively. These values indicated that our findings were reliable and robust against potential issues, such as type II errors.

## DISCUSSION

To our knowledge, this is the first large-scale MR analysis to comprehensively establish a causal relationship between cognitive performance and fractures at specific sites. Our MR study provides the initial genetic epidemiological evidence to establish a link between cognitive performance and fracture risk. Although previous observational studies have revealed this association [16], they have not definitively established a causal relationship owing to potential reverse causality and confounding factors.

Our findings underscore a significant association between cognitive function and fracture risk, highlighting disparities across the axial and appendicular skeleton. This disparity points toward a multifaceted relationship between cognitive function, biomechanics, and bone quality [17]. Specifically, the axial skeleton, comprising regions such as the hip and spine, appears to derive greater benefits from cognitive processes. These cognitive processes can profoundly influence biomechanical factors, including mechanical loading and muscle coordination, potentially affording enhanced protection to these areas through optimized biomechanical reflexes and improved postural control [18, 19]. Broadening our perspective, cognitive function [20] also plays a role in areas such as decision-making and environmental risk assessment [21–23]. This may offer protection to regions such as the ribs or pelvis by guiding individuals towards safer decisions that minimize risks [24]. In contrast, the appendicular skeleton, which is more exposed to external factors and inherently more susceptible to traumas [25], seems to be less influenced by the advantages of cognitive function.

Moving beyond just the skeletal system, it is crucial to recognize the body as a system of interconnected, but specifically interacting, parts. Known interactions, such as the gut-to-brain [26], kidney-brain [27], and gut-kidney axes [28] provide evidence for this specificity, highlighting the emerging concept of the bone-brain axis [29]. Recent research has indicated potential biological interactions between bones and the brain. For instance, Shen et al. demonstrated a mechanism for information transmission in the bone-brain axis, wherein extracellular vesicles from young chondrocytes entered the brain and improved cognitive function in mice [30].

Our results enrich the understanding of the bone-brain axis, suggesting that future research should investigate the causes and potential biological mechanisms of site specificity. Cognitive function may affect lifestyle choices that affect skeletal health, including physical activity, dietary habits, and drug use [31]. Brain-produced hormones and neurotransmitters, such as endorphins and serotonin, also affect bone metabolism [32]. Thus, our results may illuminate a crucial physiological pathway between the brain and the skeleton that warrants further exploration.

The MR method offers a near-random context for observing the effects of cognitive performance improvements on fracture risk while mitigating the influence of confounding factors. However, this method has limitations because it is predicated on several assumptions. These include instrumental variables (genetic variations) associated with the outcome only through the exposure variable, instrumental variables unrelated to any confounding factors, and no hidden direct impact between the instrumental variables and the outcome. Violations of these assumptions could result in biased estimations of causal relationships. Consequently, although our study provides robust evidence, the findings require further validation using other research methods.

Another limitation of our study is that it exclusively included European participants, leaving unknown the causal relationship between cognitive performance and fracture risk in other undefined populations. Moreover, we chose only one set of instrumental variables as the exposure factors, which may have prevented us from identifying other significant estimates. Moreover, the constraints inherent to the GWAS dataset employed within our investigation precluded the execution of MR analyses, stratified by age and sex. Additionally, the intricate mechanisms underpinning the observed associations between cognitive function and skeletal regions remain partially obscured. Further research exploring these profound linkages is warranted.

Despite these limitations, our research findings provide a fresh perspective on understanding the role of the bone-brain axis in skeletal health, which can influence healthcare policies for the older population. Unveiling the causal relationship between cognitive performance and fracture risk could influence public health policies concerning prevention and timely intervention. Enhancing cognitive performance may reduce the incidence of specific fractures and provide critical insights into potential future clinical treatments.

Our exploration of this domain is still in its early stages; however, our findings illuminate the intricate interaction between the brain and the skeletal system, presenting researchers with a novel perspective to probe the link between cerebral and skeletal health. These results could catalyze further investigations to better comprehend this relationship, potentially informing future clinical strategies. In subsequent studies, we aim to ascertain which aspects of cognitive function, such as memory, attention, and decision-making, have the strongest association with fracture risk. Furthermore, understanding whether this relationship varies with age, sex, and ethnicity is critical. With a thorough understanding of these factors, we can begin designing interventions targeted at enhancing cognitive performance to decrease the risk of fractures in the elderly. Such interventions could encompass cognitive training, nutritional adjustments, and lifestyle enhancements [33, 34].

Further research is required to elucidate the biological links between the brain and the skeletal system. This investigation may involve understanding how the brain, by influencing our behaviors and lifestyles, affects skeletal health, as well as how it directly impacts bone metabolism. This study will guide future research in this area by promoting more in-depth exploration and understanding of the complex relationship between brain and skeletal health.

## CONCLUSIONS

Our study results offer robust evidence for a causal relationship between enhanced cognitive performance and a decreased risk of fractures at specific sites. This finding suggests the potential of cognitive enhancement strategies as novel and effective approaches for fracture prevention. To fully exploit the implications of this association, further research is required to elucidate the biological pathways that connect cognitive performance and fracture susceptibility.

## MATERIALS AND METHODS

Our primary MR analysis was based on publicly accessible summary statistics (effect estimates and their standard errors) for the effects of individual SNP effect on the wrist, upper arm, shoulder, ribs, thoracic spine, lumbar spine, pelvis, femur, leg, and ankle.

### Exposure measurements

We leveraged single SNPs pertinent to cognitive performance from the UK Biobank and Cognitive Genomics Consortium (COGENT) dataset. These data comprise approximately 10 million genetic variations discovered among 257,841 individuals of European descent who also participated in a GWAS focused on educational attainment [35].

Cognitive performance was mainly evaluated in the UK Biobank using a standard measure of verbal-numerical reasoning. This involved 13 questions focused on logic and reasoning, which were created to examine fluid intelligence. In contrast, the COGENT study used an average of eight neuropsychological tests (±4 standard deviations) per sub-study. To be eligible for participation, participants had to provide at least one neuropsychological measure from a minimum of three cognitive domains.

The predominant assessments utilized across COGENT sub-studies included symbol-digit coding, number sequence retention, word recognition, category fluency, pictorial recall, lexicon, auditory recall for terms, auditory recall for narratives, letter fluency, and the path-creation test.

Our analytical approach emphasized the selection of independent SNPs that exhibited genome-wide significance (*p*<5e-8), while SNPs with *r*^2^>0.001 were excluded. In the selection process, SNPs with minimal *p*-values were prioritized as instrumental variables [36].

For each index SNP, we computed the F-statistic and *R*^2^ values, which represented the potency of the association and the proportion of explained variance by the corresponding instrumental variable, respectively.

### Outcome measurements

To bolster the statistical power to detect genetic loci, we used a liberal definition of fractures. Fracture cases were identified as individuals who had sustained fractures at any skeletal site, as corroborated by medical records, radiological evidence, and self-reported questionnaire responses. Genetic data pertinent to the fracture location utilized in the GWAS were procured from the FinnGen Consortium and UK Biobank.

The femur fracture dataset included 3983 cases and a control group of 211,460 individuals. The lumbar pelvic fracture data included 2859 cases and 212,839 controls. The upper arm-shoulder fractures data included 5824 cases and 202,866 controls. The rib-sternum-thoracic vertebral fracture data included 4070 patients and 211,861 controls. All datasets were obtained from the FinnGen Consortium (https://www.finngen.fi/en/accessresults).

We also used data from the Neale Lab, specifically for ankle fractures, which contained 4693 fracture cases and a control group of 330,853. Data on wrist fractures included 6663 cases and 328, 883 controls, whereas leg fracture data consisted of 2988 cases and 457,352 controls. These datasets were downloaded from the IEU OpenGWAS Project (https://gwas.mrcieu.ac.uk/).

Our study incorporated only meta-results from participants of European ancestry. A crucial aspect of our methodology was to ensure no overlap between the individuals present in the exposure and outcome datasets. The GWASs included in our study received approval from the relevant institutional review board, and all participants provided informed consent.

### Statistical analyses

**Figure 5:**
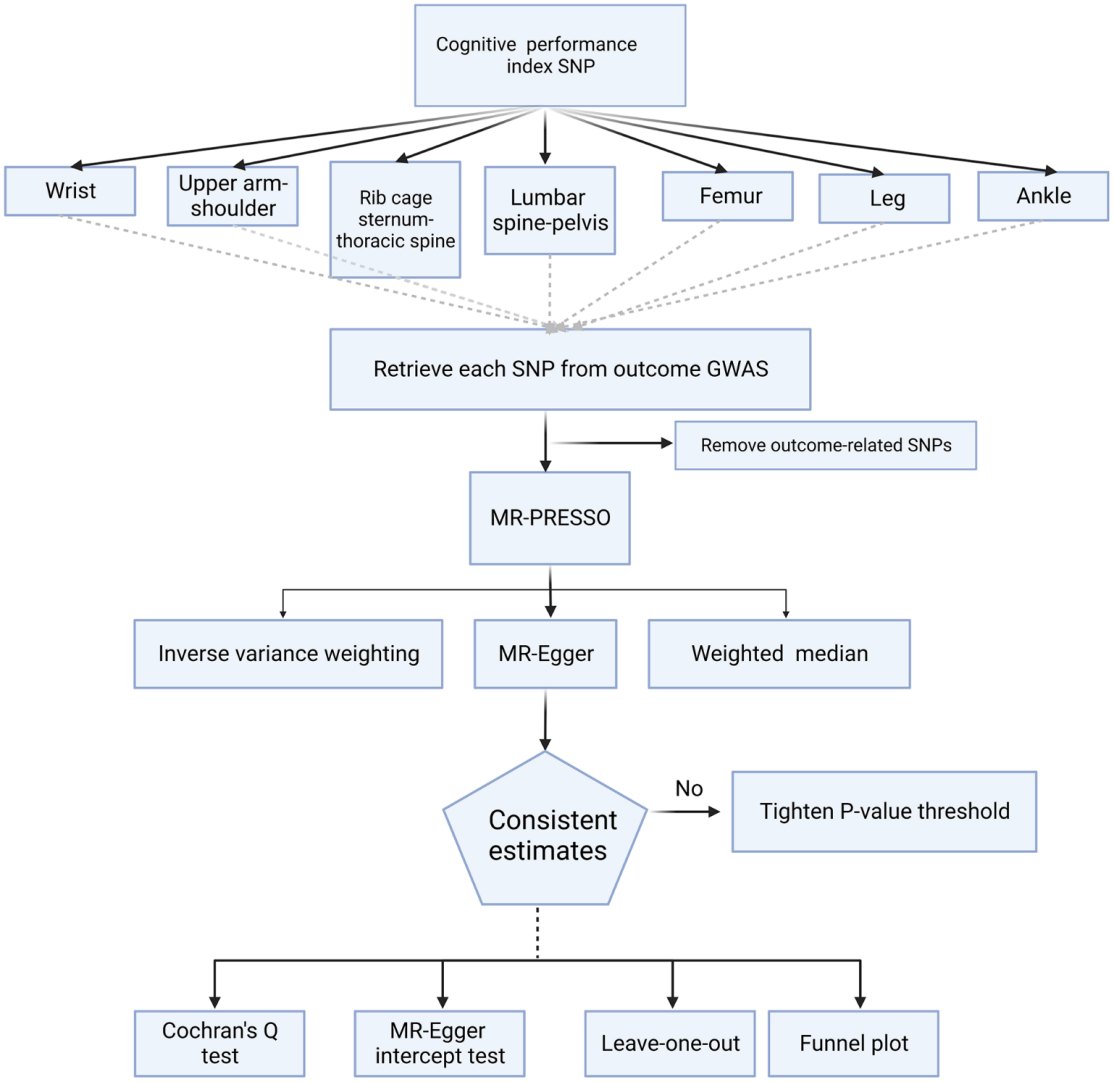
**Flowchart for the Mendelian randomization study.** This chart illustrates the process used to determine the causal relationship between cognitive performance and site-specific fracture risk. SNP, single-nucleotide polymorphism; GWAS, genome-wide association study; MR, Mendelian Randomization; MR-PRESSO Mendelian Randomization Pleiotropy RESidual Sum and Outlier

This investigation applied a suite of MR methods to ascertain the influence of cognitive performance on fracture incidence following the harmonization of effect alleles across GWASs of the two variables. Multiple MR approaches were employed to compute the estimates, including the IVW, weighted median, and MR-Egger methods. The rationale for using multiple approaches was based on the distinctive assumptions each imposes on horizontal pleiotropy (Figure 5).

The primary outcome was derived from the IVW meta-analysis of the Wald ratio for individual SNPs, based on the assumption that instrumental variables could affect the outcome solely through the exposure of interest and without any alternative pathway. Complementary to this, the MR-Egger and weighted median methods were utilized. These approaches can provide more robust estimates across a wider array of scenarios, albeit less efficiently, because of their wider confidence intervals (CIs) [37].

Sensitivity analyses have played a critical role in MR studies for detecting heterogeneity statistics and horizontal pleiotropy in MR estimates. We relied on heterogeneity markers (Cochran’s Q-derived *p* < 0.05) from the IVW method to indicate potential heterogeneity pleiotropy. The MR-Egger regression intercept served as an index for horizontal pleiotropy, with *p* < 0.05, indicating its presence. Furthermore, we employed the MR-Pleiotropy Residual Sum and Outlier (MR-PRESSO) method to evaluate and rectify the horizontal pleiotropy.

MR-PRESSO comprises three key components: (a) identification of horizontal pleiotropy, (b) rectification of horizontal pleiotropy via outlier elimination, and (c) examination of significant differences in causal estimates before and after outlier rectification. MR-PRESSO exhibits less bias and superior precision compared with IVW and MR-Egger when the percentage of horizontal pleiotropy variants decreases to <10%.

Power calculations were performed using an Internet-based application specifically designed for binary outcomes (https://shiny.cnsgenomics.com/mRnd/). Several key factors, such as a 1.25% type I error rate following multiple testing adjustments, the variance percentage (*R*^2^) in the exposure explained through genetic markers, the real impact of cognitive performance on fractures, and the ratio of cases to controls, indicated the statistical potency of our MR.

A leave-one-out analysis was employed to further assess the solidity of our MR calculations and identify whether a single SNP had an outsized impact on or distorted the estimate. We also carried out an additional assessment of potential confounders for pleiotropy using the SNP Annotator tool at http://www.phenoscanner.medschl.cam.ac.uk/upload/.

The two-sample MR (version 0.5.7) and MR-PRESSO (version 1.0) packages in R (version 4.3.0) facilitated the implementation of the analyses.
